# Crystal structure of bis­(2-methyl-1*H*-imidazol-3-ium) μ-oxalato-bis­[*n*-butyl­tri­chlorido­stannate(IV)]

**DOI:** 10.1107/S2056989016008434

**Published:** 2016-05-27

**Authors:** Mouhamadou Birame Diop, Libasse Diop, Allen G. Oliver

**Affiliations:** a Laboratoire de Chimie Minérale et Analytique, Département de Chimie, Faculté des Sciences et Techniques, Université Cheikh Anta Diop, Dakar, Senegal; b Department of Chemistry and Biochemistry, University of Notre Dame, Notre Dame, IN 46557-5670, USA

**Keywords:** crystal structure, organotin(IV) complex, oxalate, hydrogen bonds

## Abstract

The Sn^IV^ atom in the centrosymmetric anion of the title salt, (C_4_H_7_N_2_)_2_[Sn_2_(C_4_H_9_)_2_(C_2_O_4_)Cl_6_], is coordinated in a distorted octa­hedral mode by two O atoms of a bridging oxalate moiety, three Cl atoms and a C atom of an *n*-butyl group. The latter is disordered over two sets of sites in a 0.66:0.33 occupancy ratio. N—H⋯O and N—H⋯Cl hydrogen bonds involving the 2-methyl­imidazolium cation and neighbouring anions result in the formation of chains extending parallel to [001].

## Abstract 2

The Sn^IV^ atom in the centrosymmetric anion of the title salt is coordinated in a distorted octa­hedral fashion by two O atoms of the bridging oxalate moiety, a C atom of the butyl chain and three Cl atoms. The bis­(2-methyl-1*H*-imidazol-3-ium) cation forms hydrogen bonds with Cl and oxalate O atoms yielding [001] chains.

## Chemical context   

Ammonium salts of oxalatostannates(IV) with additional halogen atoms bonded within the anion are well known in the literature. Skapski *et al.* (1974 ▸) have reported the crystal structure of [(*R*_4_N)_2_][C_2_O_4_(SnCl_4_)_2_] (*R* = eth­yl) while Le Floch *et al.* (1975 ▸) have published spectroscopic studies of [(*R*_4_N)_2_][C_2_O_4_(Sn*X*_4_)_2_] (*R* = ethyl, *X* = Cl, Br, I; *R* = butyl, *X* = Br). Our group has investigated several complexes containing an oxalate group chelating an SnCl_4_ moiety or an [SnCl_3_·H_2_O]^+^ fragment, resulting in framework structures (Sow *et al.*, 2013 ▸; Diop *et al.*, 2015 ▸). In all cases, the environment around the tin(IV) atom is distorted octa­hedral.
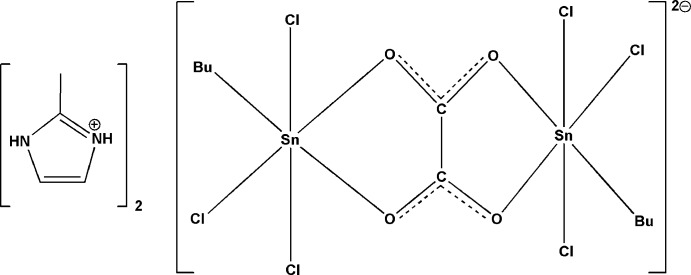


In the present communication we report on the reaction between 2-methyl-imidazolium hydrogenoxalate dihydrate and tin(IV) butyl­trichloride that yielded the title compound, (C_4_H_7_N_2_)_2_[(Sn_2_(C_4_H_9_)_2_(C_2_O_4_)Cl_6_].

## Structural commentary   

**Figure 1:**
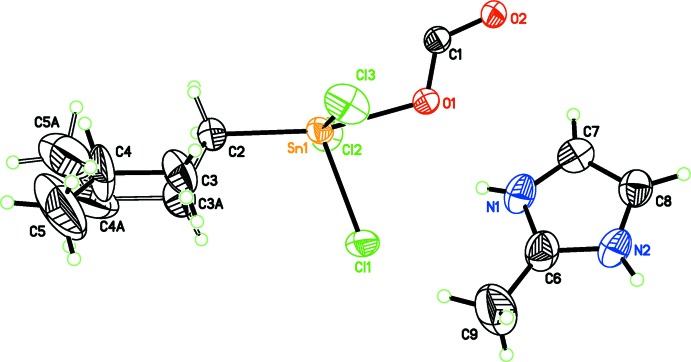
The asymmetric unit of the title compound. Displacement ellipsoids are drawn at the 50% probability level. Disordered parts of the *n*-butyl chain are shown.

**Figure 2:**
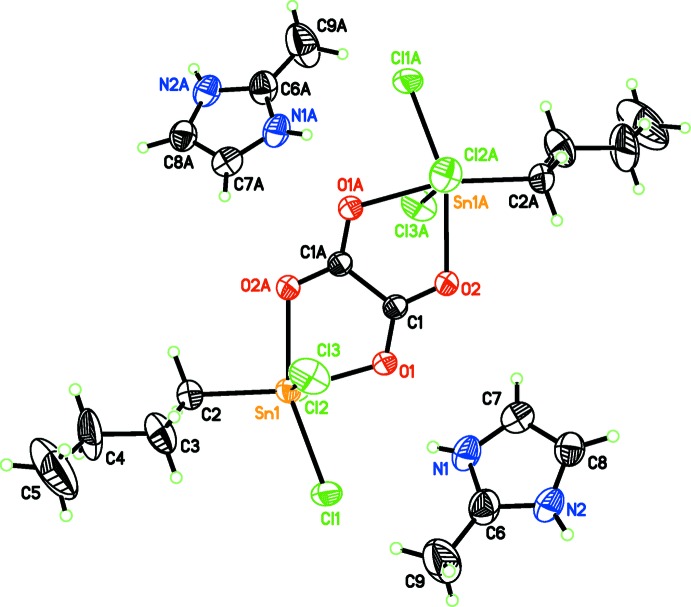
The full anion and two counter-cations in the title compound. Displacement ellipsoids are drawn at the 50% probability level. Only the major part of the disordered *n*-butyl chain is shown. [Symmetry code: (A) −*x* + 1, −*y* + 1, −*z* + 1.]

The distannate anion, [Sn_2_(C_4_H_9_)_2_(C_2_O_4_)Cl_6_]^2−^, is located about a center of symmetry and thus only one half of the mol­ecule is present in the asymmetric unit (Fig. 1 ▸). The full mol­ecule consists of a central oxalate anion bridging two SnBuCl_3_ moieties (Fig. 2 ▸) similar to the binuclear stan­nate(IV) anion reported for (Et_4_N)_2_[C_2_O_4_(SnCl_4_)_2_] (Skapski *et al.*, 1974 ▸). In addition to the bis-chelating and bridging oxalate oxygen atoms, the octa­hedral coordination sphere is completed by three chlorine atoms and the C atom of a disordered *n*-butyl group (Fig. 1 ▸). The C—O distances (Table 1 ▸) are consistent with an almost perfect π delocalization within the oxalate anion, as expected for a centrosymmetric bis-chelation. The Sn—C length is consistent with previously reported values (Table 1 ▸; Diop *et al.*, 2013 ▸). The Sn—Cl distances (Table 1 ▸) are also comparable with those in related compounds, *e.g.* in (Bu_4_N)[SnBuCl_4_] (Diop *et al.*, 2013 ▸), (Me_4_N)[C_2_O_4_SnCl_3_(H_2_O)] (Sow *et al.*, 2013 ▸) or [(methyl-2-imidazolium)][C_2_O_4_SnCl_3_(H_2_O)] (Diop *et al.*, 2015 ▸). The equatorial Sn—Cl1 bond that is coplanar with the oxalate anion is considerably shorter than the Sn—Cl2 and Sn—Cl3 bonds that are oriented axially (Fig. 2 ▸, Table 1 ▸). The Sn—O1 and Sn—O2 bond lengths are fully consistent with previously characterized examples (Sow *et al.*, 2013 ▸; Gueye *et al.*, 2014 ▸; Sarr *et al.*, 2015 ▸). Distortions from an ideal octa­hedral coordination environment are reflected in the bond angles about the Sn^IV^ atom (Table 1 ▸). Notably, the O1—Sn—O2 angle is less than 90° and the axial Cl2—Sn—Cl3 bond angle deviates considerably from an ideal of 180°.

One methyl-2-imidazolium counter-cation is also present in the asymmetric unit. As expected, the lengths of the C—N and C7—C8 bonds indicate π-delocalization in this cation (Table 1 ▸).

## Supra­molecular features   

**Figure 3:**
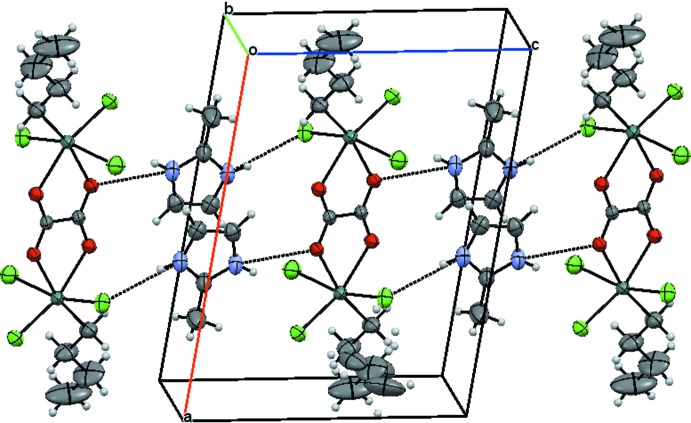
The packing of the mol­ecular components in a view approximately along [010]. N—H⋯O and N—H⋯Cl hydrogen bonds are shown as dashed lines. Displacement ellipsoids are drawn at the 50% probability level.

The imidazolium cation bridges two neighbouring [Sn_2_(C_4_H_9_)_2_(C_2_O_4_)Cl_6_]^2−^ anions through N—H⋯O and N—H⋯Cl hydrogen bonds, leading to the formation of chains extending parallel to [001] (Fig. 3 ▸, Table 2 ▸) whereby pairs of the cations are involved in this bridging motif, each alternating across the inversion center located between the cations. The chains are connected by additional C—H⋯Cl hydrogen bonds, giving a layer structure parallel to (100).

## Database survey   

A search of the Cambridge Structural Database (Version 5.37 with one update; Groom *et al.*, 2016 ▸) returned 51 different structures containing 2-methyl-1*H*-imidazol-3-ium cations and hundreds of those containing bis-chelating oxalate anions. Those of particular relevance to the title structure have been detailed above.

## Synthesis and crystallization   

Crystals of [2-methyl-1*H*-imidazol-3-ium][HC_2_O_4_·2H_2_O] (*L*) were obtained by mixing equimolar amounts of 2-methyl-imidazole with oxalic acid in water, followed by forced evaporation of the solvent at 333 K. A molar 2:1 mixture of (*L*) with SnBuCl_3_ in aceto­nitrile was allowed to react. Crystals of the title compound suitable for structural examination were obtained after slow evaporation of aceto­nitrile at room temperature.

## Refinement   

Crystal data, data collection and structure refinement details are summarized in Table 3 ▸. Hydrogen atoms were included in geometrically calculated positions with C—H = 0.98 (meth­yl) and 0.99 Å (methyl­ene), with *U*_iso_(H) = 1.5*U*_eq_(C) (meth­yl), and 1.2*U*_eq_(C) (methyl­ene). H atoms bound to N atoms within the cation were derived from difference maps and were refined freely. The *n*-butyl group was found to exhibit positional disorder, and was modelled with the peripheral three carbon atoms disordered over two sets of sites. Occupancies for these two sets were initially refined upon inspection of the refined occupancies. In the final model the occupancies were fixed at 2/3:1/3. Disordered pairs of carbon atoms (C3/C3*A*, C4/C4*A*, C5/C5*A*) were restrained to have similar atomic displacement parameters.

## Supplementary Material

Crystal structure: contains datablock(s) I. DOI: 10.1107/S2056989016008434/wm5293sup1.cif

Structure factors: contains datablock(s) I. DOI: 10.1107/S2056989016008434/wm5293Isup2.hkl

CCDC reference: 1481678

Additional supporting information:  crystallographic information; 3D view; checkCIF report

## Figures and Tables

**Table 1:** Selected geometric parameters (Å, °)

Sn1—C2	2.122 (2)	O2—C1	1.243 (2)
Sn1—O1	2.1878 (13)	O2—Sn1^i^	2.2475 (13)
Sn1—O2^i^	2.2475 (13)	N1—C6	1.313 (3)
Sn1—Cl1	2.3731 (5)	N1—C7	1.354 (3)
Sn1—Cl3	2.4460 (6)	N2—C6	1.323 (3)
Sn1—Cl2	2.4536 (5)	N2—C8	1.356 (3)
O1—C1	1.248 (2)	C7—C8	1.336 (3)
			
C2—Sn1—O1	166.44 (7)	O2^i^—Sn1—Cl3	86.17 (4)
C2—Sn1—O2^i^	92.40 (7)	Cl1—Sn1—Cl3	92.40 (2)
O1—Sn1—O2^i^	74.04 (5)	C2—Sn1—Cl2	96.58 (7)
C2—Sn1—Cl1	108.24 (6)	O1—Sn1—Cl2	82.42 (4)
O1—Sn1—Cl1	85.32 (4)	O2^i^—Sn1—Cl2	84.14 (4)
O2^i^—Sn1—Cl1	159.27 (4)	Cl1—Sn1—Cl2	91.38 (2)
C2—Sn1—Cl3	98.81 (7)	Cl3—Sn1—Cl2	162.13 (2)
O1—Sn1—Cl3	80.48 (4)		

Symmetry code: (i) 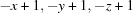.

**Table 2:** Hydrogen-bond geometry (Å, °)

*D*—H⋯*A*	*D*—H	H⋯*A*	*D*⋯*A*	*D*—H⋯*A*
N1—H1*N*⋯Cl1	0.74 (3)	2.75 (3)	3.398 (2)	147 (3)
N1—H1*N*⋯O1	0.74 (3)	2.44 (3)	2.993 (2)	133 (3)
N2—H2*N*⋯Cl2^ii^	0.77 (2)	2.43 (3)	3.187 (2)	170 (2)
C7—H7⋯Cl3^iii^	0.95	2.87	3.517 (2)	127
C9—H9*A*⋯Cl1	0.98	2.92	3.696 (3)	136

Symmetry codes: (ii) 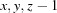; (iii) 
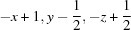
.

**Table 3:** Experimental details

Crystal data	
Chemical formula	(C_4_H_7_N_2_)_2_[Sn_2_(C_4_H_9_)_2_(C_2_O_4_)Cl_6_]
*M*_r_	818.60
Crystal system, space group	Monoclinic, *P*2_1_/*c*
Temperature (K)	200
*a*, *b*, *c* (Å)	13.4674 (5), 11.4709 (4), 10.2030 (3)
β (°)	100.453 (1)
*V* (Å^3^)	1550.03 (9)
*Z*	2
Radiation type	Mo *K*α
μ (mm^−1^)	2.16
Crystal size (mm)	0.29 × 0.18 × 0.12

Data collection	
Diffractometer	Bruker Kappa X8 APEXII
Absorption correction	Numerical (*SADABS*; Krause *et al.*, 2015 ▸)
*T*_min_, *T*_max_	0.671, 0.811
No. of measured, independent and observed [*I* > 2σ(*I*)] reflections	20211, 3868, 3490
*R*_int_	0.018
(sin θ/λ)_max_ (Å^−1^)	0.668

Refinement	
*R*[*F* ^2^ > 2σ(*F* ^2^)], *wR*(*F* ^2^), *S*	0.021, 0.051, 1.06
No. of reflections	3868
No. of parameters	192
No. of restraints	18
H-atom treatment	H atoms treated by a mixture of independent and constrained refinement
Δρ_max_, Δρ_min_ (e Å^−3^)	0.69, −0.46

Computer programs: *APEX2* and *SAINT* (Bruker, 2015 ▸), *SHELXT2014* (Sheldrick, 2015*a* ▸), *SHELXL2014* (Sheldrick, 2015*b* ▸), *XP* (Sheldrick, 2008 ▸), *CIFTAB* (Sheldrick, 2008 ▸) and *publCIF* (Westrip, 2010 ▸).

## supplementary crystallographic information

### Crystal data

**Table:**

(C_4_H_7_N_2_)_2_[Sn_2_(C_4_H_9_)_2_(C_2_O_4_)Cl_6_]	*F*(000) = 804
*M**_r_* = 818.60	*D*_x_ = 1.754 Mg m^−^^3^
Monoclinic, *P*2_1_/*c*	Mo *K*α radiation, λ = 0.71073 Å
*a* = 13.4674 (5) Å	Cell parameters from 9885 reflections
*b* = 11.4709 (4) Å	θ = 2.7–28.3°
*c* = 10.2030 (3) Å	µ = 2.16 mm^−^^1^
β = 100.453 (1)°	*T* = 200 K
*V* = 1550.03 (9) Å^3^	Block, colorless
*Z* = 2	0.29 × 0.18 × 0.12 mm

### Data collection

**Table:**

Bruker Kappa X8 APEXII diffractometer	3868 independent reflections
Radiation source: fine-focus sealed tube	3490 reflections with *I* > 2σ(*I*)
Graphite monochromator	*R*_int_ = 0.018
Detector resolution: 8.33 pixels mm^-1^	θ_max_ = 28.3°, θ_min_ = 2.4°
combination of ω and φ–scans	*h* = −17→13
Absorption correction: numerical (*SADABS*; Krause *et al.*, 2015)	*k* = −14→15
*T*_min_ = 0.671, *T*_max_ = 0.811	*l* = −13→13
20211 measured reflections	

### Refinement

**Table:**

Refinement on *F*^2^	Primary atom site location: real-space vector search
Least-squares matrix: full	Secondary atom site location: difference Fourier map
*R*[*F*^2^ > 2σ(*F*^2^)] = 0.021	Hydrogen site location: mixed
*wR*(*F*^2^) = 0.051	H atoms treated by a mixture of independent and constrained refinement
*S* = 1.06	*w* = 1/[σ^2^(*F*_o_^2^) + (0.0236*P*)^2^ + 0.9516*P*] where *P* = (*F*_o_^2^ + 2*F*_c_^2^)/3
3868 reflections	(Δ/σ)_max_ = 0.022
192 parameters	Δρ_max_ = 0.69 e Å^−^^3^
18 restraints	Δρ_min_ = −0.46 e Å^−^^3^

### Special details

**Table:**

Geometry. All esds (except the esd in the dihedral angle between two l.s. planes) are estimated using the full covariance matrix. The cell esds are taken into account individually in the estimation of esds in distances, angles and torsion angles; correlations between esds in cell parameters are only used when they are defined by crystal symmetry. An approximate (isotropic) treatment of cell esds is used for estimating esds involving l.s. planes.
Refinement. Disorder in the n-butyl chain was modeled over two sites. Occupancies were initially refined and subsequently set to 0.66667:0.33333. Carbon atoms were refined with anisotropic atomic displacement parameters and the disordered carbon atoms were restrained to have similar displacement parameters.

### Fractional atomic coordinates and isotropic or equivalent isotropic displacement parameters (Å^2^)

**Table:**

	*x*	*y*	*z*	*U*_iso_*/*U*_eq_	Occ. (<1)
Sn1	0.71591 (2)	0.52312 (2)	0.53372 (2)	0.03131 (5)	
Cl1	0.81275 (4)	0.42161 (6)	0.39795 (5)	0.04767 (13)	
Cl2	0.71227 (4)	0.35142 (5)	0.67619 (5)	0.04538 (12)	
Cl3	0.66628 (5)	0.67332 (6)	0.36433 (6)	0.05395 (15)	
O1	0.58579 (10)	0.43651 (13)	0.41329 (12)	0.0341 (3)	
O2	0.41920 (10)	0.42053 (12)	0.38137 (13)	0.0339 (3)	
C1	0.50131 (14)	0.45924 (16)	0.44091 (17)	0.0296 (4)	
C2	0.81592 (17)	0.6238 (2)	0.6744 (2)	0.0465 (5)	
H2A	0.7753	0.6723	0.7251	0.056*	0.6667
H2B	0.8569	0.5700	0.7383	0.056*	0.6667
H2C	0.7810	0.6968	0.6911	0.056*	0.3333
H2D	0.8300	0.5800	0.7594	0.056*	0.3333
C3	0.8844 (5)	0.7002 (5)	0.6182 (8)	0.0621 (16)	0.6667
H3A	0.8439	0.7537	0.5534	0.075*	0.6667
H3B	0.9263	0.6520	0.5689	0.075*	0.6667
C4	0.9514 (6)	0.7697 (7)	0.7186 (9)	0.096 (3)	0.6667
H4A	0.9091	0.8112	0.7734	0.115*	0.6667
H4B	0.9955	0.7154	0.7783	0.115*	0.6667
C5	1.0148 (5)	0.8544 (6)	0.6693 (11)	0.147 (4)	0.6667
H5A	1.0609	0.8144	0.6202	0.221*	0.6667
H5B	1.0539	0.8973	0.7444	0.221*	0.6667
H5C	0.9725	0.9090	0.6097	0.221*	0.6667
C3A	0.9164 (10)	0.6557 (15)	0.6353 (18)	0.089 (5)	0.3333
H3C	0.9050	0.7118	0.5603	0.107*	0.3333
H3D	0.9483	0.5850	0.6057	0.107*	0.3333
C4A	0.9967 (9)	0.7180 (13)	0.772 (2)	0.125 (6)	0.3333
H4C	0.9875	0.6829	0.8578	0.150*	0.3333
H4D	1.0686	0.7138	0.7634	0.150*	0.3333
C5A	0.9606 (11)	0.8257 (19)	0.7572 (18)	0.115 (5)	0.3333
H5D	1.0101	0.8801	0.8061	0.173*	0.3333
H5E	0.8976	0.8304	0.7921	0.173*	0.3333
H5F	0.9476	0.8462	0.6624	0.173*	0.3333
N1	0.61980 (17)	0.41626 (18)	0.13231 (19)	0.0462 (5)	
H1N	0.644 (2)	0.434 (3)	0.201 (3)	0.059 (9)*	
N2	0.60535 (16)	0.37133 (18)	−0.07045 (18)	0.0463 (5)	
H2N	0.6237 (19)	0.366 (2)	−0.137 (2)	0.044 (7)*	
C6	0.6683 (2)	0.4165 (2)	0.0316 (2)	0.0487 (5)	
C7	0.52646 (18)	0.36986 (19)	0.0958 (2)	0.0438 (5)	
H7	0.4773	0.3595	0.1508	0.053*	
C8	0.51718 (18)	0.34151 (19)	−0.0328 (2)	0.0438 (5)	
H8A	0.4600	0.3072	−0.0873	0.053*	
C9	0.7714 (3)	0.4571 (4)	0.0320 (3)	0.0894 (12)	
H9A	0.7993	0.4905	0.1193	0.134*	
H9B	0.7707	0.5166	−0.0370	0.134*	
H9C	0.8134	0.3913	0.0140	0.134*	

### Atomic displacement parameters (Å^2^)

**Table:**

	*U*^11^	*U*^22^	*U*^33^	*U*^12^	*U*^13^	*U*^23^
Sn1	0.02611 (7)	0.04132 (8)	0.02698 (7)	−0.00213 (5)	0.00611 (5)	−0.00174 (5)
Cl1	0.0358 (3)	0.0706 (4)	0.0385 (3)	0.0073 (2)	0.0116 (2)	−0.0100 (2)
Cl2	0.0549 (3)	0.0459 (3)	0.0359 (2)	−0.0024 (2)	0.0097 (2)	0.0052 (2)
Cl3	0.0489 (3)	0.0611 (4)	0.0522 (3)	0.0034 (3)	0.0101 (3)	0.0221 (3)
O1	0.0282 (7)	0.0459 (7)	0.0289 (6)	−0.0013 (6)	0.0074 (5)	−0.0078 (6)
O2	0.0286 (7)	0.0432 (8)	0.0304 (6)	−0.0032 (6)	0.0065 (5)	−0.0085 (6)
C1	0.0300 (9)	0.0354 (9)	0.0243 (8)	−0.0013 (7)	0.0073 (7)	−0.0005 (7)
C2	0.0365 (11)	0.0579 (14)	0.0445 (11)	−0.0075 (10)	0.0058 (9)	−0.0150 (10)
C3	0.045 (3)	0.059 (3)	0.078 (3)	−0.020 (2)	0.000 (3)	0.002 (3)
C4	0.077 (5)	0.080 (5)	0.121 (6)	−0.043 (4)	−0.009 (4)	−0.041 (4)
C5	0.061 (4)	0.082 (4)	0.283 (12)	−0.032 (3)	−0.009 (5)	−0.007 (6)
C3A	0.050 (8)	0.110 (13)	0.111 (12)	−0.031 (7)	0.024 (7)	−0.061 (10)
C4A	0.053 (7)	0.094 (10)	0.233 (19)	0.011 (6)	0.042 (9)	−0.020 (11)
C5A	0.058 (8)	0.169 (18)	0.122 (13)	−0.011 (10)	0.027 (8)	−0.018 (12)
N1	0.0598 (13)	0.0507 (11)	0.0279 (9)	−0.0053 (9)	0.0073 (8)	−0.0023 (8)
N2	0.0590 (12)	0.0515 (11)	0.0290 (9)	−0.0022 (9)	0.0097 (8)	−0.0031 (8)
C6	0.0551 (14)	0.0579 (14)	0.0330 (10)	−0.0088 (11)	0.0080 (9)	0.0010 (9)
C7	0.0513 (13)	0.0393 (11)	0.0425 (11)	0.0014 (9)	0.0132 (10)	0.0031 (9)
C8	0.0495 (12)	0.0363 (10)	0.0443 (11)	−0.0019 (9)	0.0050 (10)	−0.0012 (9)
C9	0.063 (2)	0.153 (4)	0.0526 (17)	−0.038 (2)	0.0123 (14)	−0.0002 (19)

### Geometric parameters (Å, º)

**Table:**

Sn1—C2	2.122 (2)	C5—H5C	0.9800
Sn1—O1	2.1878 (13)	C3A—C4A	1.76 (2)
Sn1—O2^i^	2.2475 (13)	C3A—H3C	0.9900
Sn1—Cl1	2.3731 (5)	C3A—H3D	0.9900
Sn1—Cl3	2.4460 (6)	C4A—C5A	1.33 (2)
Sn1—Cl2	2.4536 (5)	C4A—H4C	0.9900
O1—C1	1.248 (2)	C4A—H4D	0.9900
O2—C1	1.243 (2)	C5A—H5D	0.9800
O2—Sn1^i^	2.2475 (13)	C5A—H5E	0.9800
C1—C1^i^	1.531 (3)	C5A—H5F	0.9800
C2—C3	1.463 (7)	N1—C6	1.313 (3)
C2—C3A	1.524 (16)	N1—C7	1.354 (3)
C2—H2A	0.9900	N1—H1N	0.74 (3)
C2—H2B	0.9900	N2—C6	1.323 (3)
C2—H2C	0.9900	N2—C8	1.356 (3)
C2—H2D	0.9900	N2—H2N	0.77 (2)
C3—C4	1.471 (9)	C6—C9	1.465 (4)
C3—H3A	0.9900	C7—C8	1.336 (3)
C3—H3B	0.9900	C7—H7	0.9500
C4—C5	1.443 (11)	C8—H8A	0.9500
C4—H4A	0.9900	C9—H9A	0.9800
C4—H4B	0.9900	C9—H9B	0.9800
C5—H5A	0.9800	C9—H9C	0.9800
C5—H5B	0.9800		
			
C2—Sn1—O1	166.44 (7)	C4—C5—H5B	109.5
C2—Sn1—O2^i^	92.40 (7)	H5A—C5—H5B	109.5
O1—Sn1—O2^i^	74.04 (5)	C4—C5—H5C	109.5
C2—Sn1—Cl1	108.24 (6)	H5A—C5—H5C	109.5
O1—Sn1—Cl1	85.32 (4)	H5B—C5—H5C	109.5
O2^i^—Sn1—Cl1	159.27 (4)	C2—C3A—C4A	109.7 (11)
C2—Sn1—Cl3	98.81 (7)	C2—C3A—H3C	109.7
O1—Sn1—Cl3	80.48 (4)	C4A—C3A—H3C	109.7
O2^i^—Sn1—Cl3	86.17 (4)	C2—C3A—H3D	109.7
Cl1—Sn1—Cl3	92.40 (2)	C4A—C3A—H3D	109.7
C2—Sn1—Cl2	96.58 (7)	H3C—C3A—H3D	108.2
O1—Sn1—Cl2	82.42 (4)	C5A—C4A—C3A	97.3 (16)
O2^i^—Sn1—Cl2	84.14 (4)	C5A—C4A—H4C	112.3
Cl1—Sn1—Cl2	91.38 (2)	C3A—C4A—H4C	112.3
Cl3—Sn1—Cl2	162.13 (2)	C5A—C4A—H4D	112.3
C1—O1—Sn1	116.64 (12)	C3A—C4A—H4D	112.3
C1—O2—Sn1^i^	114.80 (11)	H4C—C4A—H4D	109.9
O2—C1—O1	125.55 (17)	C4A—C5A—H5D	109.5
O2—C1—C1^i^	117.2 (2)	C4A—C5A—H5E	109.5
O1—C1—C1^i^	117.2 (2)	H5D—C5A—H5E	109.5
C3—C2—Sn1	115.4 (3)	C4A—C5A—H5F	109.5
C3A—C2—Sn1	116.0 (6)	H5D—C5A—H5F	109.5
C3—C2—H2A	108.4	H5E—C5A—H5F	109.5
Sn1—C2—H2A	108.4	C6—N1—C7	110.7 (2)
C3—C2—H2B	108.4	C6—N1—H1N	122 (2)
Sn1—C2—H2B	108.4	C7—N1—H1N	126 (2)
H2A—C2—H2B	107.5	C6—N2—C8	110.15 (19)
C3A—C2—H2C	108.3	C6—N2—H2N	117.8 (19)
Sn1—C2—H2C	108.3	C8—N2—H2N	132.1 (19)
C3A—C2—H2D	108.3	N1—C6—N2	106.0 (2)
Sn1—C2—H2D	108.3	N1—C6—C9	127.2 (2)
H2C—C2—H2D	107.4	N2—C6—C9	126.7 (2)
C2—C3—C4	113.7 (6)	C8—C7—N1	106.4 (2)
C2—C3—H3A	108.8	C8—C7—H7	126.8
C4—C3—H3A	108.8	N1—C7—H7	126.8
C2—C3—H3B	108.8	C7—C8—N2	106.6 (2)
C4—C3—H3B	108.8	C7—C8—H8A	126.7
H3A—C3—H3B	107.7	N2—C8—H8A	126.7
C5—C4—C3	116.7 (8)	C6—C9—H9A	109.5
C5—C4—H4A	108.1	C6—C9—H9B	109.5
C3—C4—H4A	108.1	H9A—C9—H9B	109.5
C5—C4—H4B	108.1	C6—C9—H9C	109.5
C3—C4—H4B	108.1	H9A—C9—H9C	109.5
H4A—C4—H4B	107.3	H9B—C9—H9C	109.5
C4—C5—H5A	109.5		
			
Sn1^i^—O2—C1—O1	177.81 (15)	C7—N1—C6—N2	0.7 (3)
Sn1^i^—O2—C1—C1^i^	−1.5 (3)	C7—N1—C6—C9	−178.6 (3)
Sn1—O1—C1—O2	178.19 (15)	C8—N2—C6—N1	−0.6 (3)
Sn1—O1—C1—C1^i^	−2.5 (3)	C8—N2—C6—C9	178.7 (3)
Sn1—C2—C3—C4	179.1 (5)	C6—N1—C7—C8	−0.5 (3)
C2—C3—C4—C5	−174.8 (6)	N1—C7—C8—N2	0.1 (3)
Sn1—C2—C3A—C4A	−170.7 (8)	C6—N2—C8—C7	0.3 (3)
C2—C3A—C4A—C5A	−83.0 (15)		

Symmetry code: (i) −*x*+1, −*y*+1, −*z*+1.

### Hydrogen-bond geometry (Å, º)

**Table:**

*D*—H···*A*	*D*—H	H···*A*	*D*···*A*	*D*—H···*A*
N1—H1*N*···Cl1	0.74 (3)	2.75 (3)	3.398 (2)	147 (3)
N1—H1*N*···O1	0.74 (3)	2.44 (3)	2.993 (2)	133 (3)
N2—H2*N*···Cl2^ii^	0.77 (2)	2.43 (3)	3.187 (2)	170 (2)
C7—H7···Cl3^iii^	0.95	2.87	3.517 (2)	127
C9—H9*A*···Cl1	0.98	2.92	3.696 (3)	136

Symmetry codes: (ii) *x*, *y*, *z*−1; (iii) −*x*+1, *y*−1/2, −*z*+1/2.
